# Impact of dementia and mild cognitive impairment on bone health in older people

**DOI:** 10.1007/s40520-024-02871-y

**Published:** 2024-12-27

**Authors:** Elizabeth M. Curtis, Mario Miguel, Claire McEvoy, Andrea Ticinesi, Carla Torre, Nasser Al-Daghri, Majed Alokail, Ewa Bałkowiec-Iskra, Olivier Bruyère, Nansa Burlet, Etienne Cavalier, Francesca Cerreta, Patricia Clark, Antonio Cherubini, Cyrus Cooper, Patrizia D’Amelio, Nicholas Fuggle, Celia Gregson, Philippe Halbout, John A. Kanis, Jean Kaufman, Andrea Laslop, Stefania Maggi, Andrea Maier, Radmila Matijevic, Eugene McCloskey, Sif Ormarsdóttir, Concha Prieto Yerro, Régis P. Radermecker, Yves Rolland, Andrea Singer, Nicola Veronese, René Rizzoli, Jean-Yves Reginster, Nicholas C. Harvey

**Affiliations:** 1 MRC Lifecourse Epidemiology Centre, University of Southampton, Southampton, UK; 2 NIHR Southampton Biomedical Research Centre, University of Southampton, Southampton, UK; 3 Centro de Estudos Egas Moniz, Faculdade de Medicina da Universidade de Lisboa, Lisbon, Portugal; 4 Centre for Public Health, School of Medicine, Dentistry and Biomedical Sciences, Queen’s University, Belfast, UK; 5 Department of Medicine and Surgery, University of Parma, Parma, Italy; 6 Azienda Ospedaliero-Universitaria Di Parma, Parma, Italy; 7 Faculdade de Farmácia, Universidade de Lisboa, Avenida Professor Gama Pinto, 1649-003 Lisbon, Portugal; 8 Laboratory of Systems Integration Pharmacology, Clinical and Regulatory Science, Research Institute for Medicines of the University of Lisbon (iMED.ULisboa), Avenida Professor Gama Pinto, 1649-003 Lisbon, Portugal; 9 Chair for Biomarkers of Chronic Diseases, Biochemistry Department, College of Science, King Saud University, 11451 Riyadh, Kingdom of Saudi Arabia; 10 Biochemistry Department, College of Science, KSU, Riyadh, Kingdom of Saudi Arabia; 11 Department of Experimental and Clinical Pharmacology, Medical University of Warsaw, Warsaw, Poland; 12 The Office for Registration of Medicinal Products, Medical Devices and Biocidal Products & CHMP, SAWP, CNSWP, PCWP, ETF (European Medicines Agency) Member, Warsaw, Poland; 13 Research Unit in Public Health, Epidemiology and Health Economics, University of Liège, Liège, Belgium; 14 Department of Physical Activity and Rehabilitation Sciences, University of Liège, Liège, Belgium; 15 Research Unit in Epidemiology, University of Liege, Liège, Belgium; 16 Department of Clinical Chemistry, CIRM, University of Liège, CHU de Liège, Liège, Belgium; 17 Digital Health and Geriatrics, European Medicines Agency, Amsterdam, The Netherlands; 18 Clinical Epidemiology Unit, Hospital Infantil Federico Gómez-Facultad de Medicina, National Autonomous University of Mexico (UNAM), Mexico City, Mexico; 19 Geriatria, Accettazione Geriatrica e Centro di ricerca per l’invecchiamento, IRCCS INRCA Istituto Nazionale di Ricovero e Cura per Anziani, Ancona, Italy; 20 Department of Clinical and Molecular Sciences, Università Politecnica delle Marche, Ancona, Italy; 21 Department of Medicine, Service of Geriatric Medicine & Geriatric Rehabilitation, University of Lausanne Hospital, University of Lausanne, Lausanne, Switzerland; 22 Musculoskeletal Research Unit, Bristol Medical School, Learning and Research Building, University of Bristol, Southmead Hospital, Bristol, BS10 5NB UK; 23 The Health Research Unit of Zimbabwe (THRU ZIM), The Biomedical Research and Training Institute, Harare, Zimbabwe; 24 International Osteoporosis Foundation, Nyon, Switzerland; 25 Mary McKillop Institute for Health Research, Australian Catholic University, Melbourne, Australia; 26 Centre for Metabolic Bone Diseases, University of Sheffield, Sheffield, UK; 27 Department of Endocrinology, Ghent University Hospital, Ghent, Belgium; 28 Scientific Office, Austrian Medicines and Medical Devices Agency, Vienna, Austria; 29 CNR Aging Branch-IN, Padua, Italy; 30 Healthy Longevity Translational Research Program, Yong Loo Lin School of Medicine, National University of Singapore, Singapore, 117596 Singapore; 31 Department of Human Movement Sciences, at AgeAmsterdam, Faculty of Behavioural and Movement Sciences, Vrije Universiteit Amsterdam, Amsterdam Movement Sciences, Amsterdam, The Netherlands; 32 Faculty of Medicine in Novi Sad, University of Novi Sad, Novi Sad, Serbia; 33 Mellanby Centre for Musculoskeletal Research, Division of Clinical Medicine, School of Medicine and Population Health, University of Sheffield, Sheffield, UK; 34 MRC Versus Arthritis Centre for Integrated Research in Musculoskeletal Ageing, University of Sheffield, Sheffield, UK; 35 Medicine Assessment and Licencing, Icelandic Medicines Agency, Reykjavik, Iceland; 36 Agencia Española de Medicamentos y Productos Sanitarios, Madrid, Spain; 37 Department of Diabetes, Nutrition and Metabolic Disorders, Clinical Pharmacology, University of Liege, CHU de Liège, Liège, Belgium; 38 HealthAge, CHU Toulouse, CERPOP UMR 1295, Inserm, Université Paul Sabatier, Toulouse, France; 39 Departments of Obstetrics & Gynecology and Medicine, MedStar Georgetown University Hospital, Washington, DC USA; 40 Department of Internal Medicine, Geriatrics Section, University of Palermo, Palermo, Italy; 41 Geneva University Hospitals and Faculty of Medicine, Geneva, Switzerland; 42 Protein Research Chair, Biochemistry Department, College of Science, King Saud University, Riyadh, Kingdom of Saudi Arabia

**Keywords:** Osteoporosis, Epidemiology, Cognitive impairment, Dementia, Bone mineral density, Fracture

## Abstract

Mild cognitive impairment, dementia and osteoporosis are common diseases of ageing and, with the increasingly ageing global population, are increasing in prevalence. These conditions are closely associated, with shared risk factors, common underlying biological mechanisms and potential direct causal pathways. In this review, the epidemiological and mechanistic links between mild cognitive impairment, dementia and skeletal health are explored. Discussion will focus on how changes in brain and bone signalling can underly associations between these conditions, and will consider the molecular and cellular drivers in the context of inflammation and the gut microbiome. There is a complex interplay between nutritional changes, which may precede or follow the onset of mild cognitive impairment (MCI) or dementia, and bone health. Polypharmacy is common in patients with MCI or dementia, and there are difficult prescribing decisions to be made due to the elevated risk of falls associated with many drugs used for associated problems, which can consequently increase fracture risk. Some medications prescribed for cognitive impairment may directly impact bone health. In addition, patients may have difficulty remembering medication without assistance, meaning that osteoporosis drugs may be prescribed but not taken. Cognitive impairment may be improved or delayed by physical activity and exercise, and there is evidence for the additional benefits of physical activity on falls and fractures. Research gaps and priorities with the aim of reducing the burden of osteoporosis and fractures in people with MCI or dementia will also be discussed.

## Introduction

Both osteoporosis and dementia (or mild cognitive impairment) are common conditions, generally occurring in older age. Populations worldwide are ageing and, indeed, according to the United Nations (UN) Decade of Healthy Ageing report (2021–2030), “longer lives are one of humanity's greatest achievements. But longer lives are not yet healthier lives for all” [1]. Adding quality years to people’s lives, lived in good health, maintaining capacity and preventing dependency on carers is one of the greatest challenges of modern medicine and society; reducing dementia, and also fractures as a consequence of poor bone health would have a huge impact.

With increasing lifespan, the gap between life expectancy and healthy life expectancy is increasing. According to global estimates, in the year 2000 at age 60 years, the gap between healthy life expectancy and actual life expectancy was 4.1 years for men, and 5.3 years for women. By the year 2019, this gap increased to 4.7 years for men and 6.0 years for women [1, 2]. The World Health Organisation (WHO), as part of the “Decade of Healthy Ageing”, have mandated tracking progress in reducing this gap between healthy years lived (healthspan) and actual years lived (lifespan) over the coming years, with reporting at both national and subnational levels taking place in 2023, 2026, 2029 and 2030. Key to the WHO and United Nations (UN) Decade of Healthy Ageing are the concepts of the functional ability of an individual and their intrinsic capacity. It is evident that locomotor and cognitive abilities are critical to enable a person to maintain functional ability, and indeed form part of the UN integrated care for older people (ICOPE) Assessment Framework for person-centred assessment pathways, aimed at improving integrated care for older people [3, 4].

## Dementia and mild cognitive impairment

### Definitions

Dementia (or major neurocognitive disorder, as it is termed in the Diagnostic and Statistical Manual of Mental Disorders (DSM-5^®^) [5] is an umbrella term for several (mostly progressive) irreversible neurological diseases of the brain, affecting memory, other cognitive abilities and behaviour, which interfere significantly with the ability to maintain autonomy in the activities of daily living [6]. Alzheimer’s disease (AD) is the most common form of dementia in high income settings and may contribute to 60% to 80% of cases, although figures vary according to the method of diagnosis and to the population studied. Other major forms include cerebrovascular dementia, Lewy body dementia and a group of diseases that contribute to frontal temporal dementia; there is commonly overlap between these different subtypes. Many studies focusing on links between dementia and fracture or osteoporosis are limited to patients with an AD diagnosis, whilst others have used cognitive scoring, which may detect dementia at an early stage.

Mild cognitive impairment (MCI) (or mild neurocognitive disorder [5]) is more common (its incidence may be twice as high as dementia [7]) and reflects a noticeable decrement in cognitive functioning that goes beyond normal changes seen in ageing, and represents a state of cognitive function between normal ageing and dementia. It is objectively defined on neurocognitive assessment and occurs in the absence of significant impairment of the instrumental activities of daily living [8]. The impairment covers the domains of learning, memory, attention and reasoning. MCI can be caused by a variety of different health problems which may or may not be attributable to a specific dementia diagnosis and is a disorder that may or may not progress to dementia [9]. There is a continuum across loss of higher brain functions, from early MCI through to late MCI, to mild, moderate and severe dementia with different medicines indicated for different stages. Balance and reaction time, executive function (such as the ability to pay attention, organise, plan, and/or prioritise physical tasks) and memory are affected differently between individuals and between dementia types, which may all impact upon fall and fracture risk.

### Burden of disease

Dementia is currently the seventh leading cause of death and one of the major causes of disability and dependency among older people globally [6]. According to the WHO in 2024, over 55 million people have dementia worldwide, over 60% of whom live in lower on middle income countries where there are nearly 10 million new cases per year. By 2050, it is anticipated that 135 million people will have dementia worldwide, increasing slightly less than two fold in Europe, somewhat more than twofold in North America, threefold in Asia and fourfold in Latin America and Africa [10, 11]. Dementia has a huge economic impact, costing economies globally 1.3 trillion US dollars. Approximately 50% of these costs are attributable to care provided by informal carers (e.g. family members and close friends), who provide on average 5 h of care and supervision per day. Lower income countries account for just under 1% of the total worldwide costs (and 14% of the dementia prevalence), and higher income countries for 89% of the costs (and 46% of the dementia prevalence). Such disparities are likely accounted for by the lower diagnosis rate, limited therapeutic options available and the underutilisation of the existing evidence-based interventions in low income countries [11].

In its report, the WHO states that women are disproportionately affected by dementia, both directly (they experience higher associated disability-adjusted life years and mortality), and indirectly, as they provide 70% of care hours for people living with dementia [6]. In the UK 65% of people with dementia are female, and dementia has been the leading cause of death in women since 2011 [12]. Ethnic differences are also observed, with Black people in the UK, and African Americans having a higher dementia incidence [13]. A UK based study of General Practice electronic records (Clinical Practice Research Datalink, CPRD) showed that dementia incidence was higher in Black than White people (Incidence Rate Ratio 1.22, 95% CI 1.15–1.30). South Asian and Black people with dementia also had a younger age of death than White participants (mean difference for South Asian participants − 2.97 years, (95% CI − 3.41 to − 2.53); and Black participants − 2.66 years, (95% CI − 3.08 to − 2.24) [14].

## Osteoporosis and high fracture risk

### Definition and epidemiology

Osteoporosis is a disease of the skeleton, characterised by micro-architectural deterioration of bone tissue and loss of bone mass. Osteoporosis (meaning ‘porous bone’) increases bone fragility and susceptibility to fracture [15]. The operational definition of osteoporosis (1994 World Health Organisation Definition) is predicated on the measurement of bone mineral density (BMD) (via Dual-energy X-ray Absorptiometry (DXA)) [16], with osteoporosis defined as 2.5 standard deviations below the healthy young adult female mean [17]. BMD varies across the lifecourse, reaching a peak in early adulthood during the third to fourth decade, plateauing in middle life, and then declining from around the age of 50 years. The incidence of major osteoporotic fracture types (e.g. hip, vertebral, proximal humerus, distal radius) increases with age, with a near exponential increase in hip fracture incidence in men and women beyond 75 years [18, 19] with the median age for hip fracture well above the age of 80 years in many countries [20]. Similar patterns of increasing incidence are observed for vertebral fractures in other studies [21]. Fragility fractures are also associated with substantial increases in mortality [22, 23].

It is important to consider that whilst a T score below -2.5 is the WHO definition of osteoporosis, and low BMD confers an increased risk of fracture, the majority of fractures occur in postmenopausal women and older men without a densitometric diagnosis of osteoporosis, or in those with osteopenia [24, 25]. Aside from the natural decreases in BMD seen in older people, there are a variety of other reasons why they may be at greater fracture risk, with factors such as decreased mobility, increased risk of falls, and the adjunctive effects of comorbidities (including dementia and cardiovascular disease) [26, 27]. Indeed, a recent study in nursing home populations (a frail and high fracture risk group) identified fracture risks which are underestimated by current approaches such as FRAX^®^, QFracture or the GARVAN calculator [28, 29].

### Burden of disease

The number of individuals aged 50 years or older at high risk of osteoporotic fracture worldwide was estimated at 158 million in 2010 and is set to double to around 319 million by 2040 with the biggest increase in population at risk seen in Asia and Africa [20]. It is predicted that the majority of hip fractures in Asia will occur in China, where the incidence of hip fracture will rise from 411,000 in 2015 to an estimate of more than 1 million in 2050. Indeed, huge variability in hip fracture rates worldwide are observed, with a greater than tenfold variation in hip fracture risk between countries [30]. Data from African populations are particularly sparse, with a lack of equitable access to diagnostic and treatment options to reduce the risk of fragility fractures and subsequent disability [31]. In Europe alone, the annual cost of managing fragility fractures is estimated at 56.9 billion Euros (2017) and set to increase by 27% by 2030; health and social systems even in high-income countries are ill-equipped to cope with such an increase in demand for both post fracture and dementia care.

In the oldest age groups, most likely to suffer from co-existent dementia or mild cognitive impairment alongside osteoporosis, there is often undertreatment of those requiring osteoporosis medication. For example, in the Newcastle 85 + cohort, of 259 older adults (mean age 85.5 years) who were identified as requiring treatment for osteoporosis (via fracture risk calculation), only 74 (28.6%) were receiving osteoporosis medication [32]. In this older cohort, the treatment gap (between those in whom treatment is recommended and in those actually treated) was 71.4%, higher than the UK national average of 66%, emphasising the neglect that the oldest age groups suffer when it comes to osteoporosis care [33]. Considerations for the management of osteoporosis in the oldest old were set out by European Society for Clinical and Economic Aspects of Osteoporosis, Osteoarthritis and Musculoskeletal Diseases (ESCEO), but evidence relating specifically to this population remains sparse [27, 34].

## Associations between cognitive impairment, bone mineral density and fracture risk

When considering the epidemiological evidence underlying the association between cognitive impairment and bone health, it is important to differentiate between studies examining associations between cognitive impairment and BMD, versus fracture (which also is likely to involve BMD independent mechanisms, for example increased falls risk).

The incidence of falls and fractures in people with dementia is known to be high [35], for example, in a representative UK study of 8036 people with dementia, the incidence of falls was 31.1% (125 per 1000 person years) and fractures, 17% (65.5 per 1000 person years) [36]. The authors found that the predictors of falls were increased age, female sex, physical health problems, a previous fall or fracture, the presence of vascular dementia versus AD, higher levels of neighbourhood deprivation, living alone and social determinants of health such as poor living conditions; interestingly, ethnic minority status (e.g. Caribbean or Asian ethnicity) was protective of falls in older people [36]. Unsurprisingly, there is evidence of an increased risk of all fractures in patients with dementia, with poorer health outcomes (including physical performance measures), increased social care needs and mortality [37–42]; indeed, a meta-analysis of five cohort studies on hip fracture covering over 137,000 participants, showed that AD was associated with a 2.5-fold increased risk of hip fractures [43].

In a Finnish nationwide registry-based cohort, in which verified AD cases were matched with healthy controls, those with AD experienced double the risk of hip fractures. The risk increase for fracture was larger in men than women when age groups were pooled, and highest in those who were youngest when AD was diagnosed [37]. Adjustment for health status, psychotropic drugs and bisphosphonate use failed to weaken the associations between Alzheimer's disease and hip fracture. A Korean study using health insurance data also showed that the greatest risk of fracture was noted earliest in the dementia disease course, perhaps because of early balance and gait disturbances [44]. In over 13,500 individuals with early-onset dementia (EOD) compared with healthy controls (age 50–64, identified from the National Database of Health Insurance Claims and Specific Health Checkups of Japan), EOD was associated with an increased risk of hip fractures (adjusted odds ratio, 95% confidence interval: 8.79, 7.37–10.48), vertebral fractures (1.73, 1.48–2.01), and major osteoporotic fractures (2.05, 1.83–2.30) over 3 years [45]. In general, however, the evidence for associations between dementia and non-hip fractures is not as robust. A Taiwanese study with three-year follow up indicated a doubling of hip fracture risk, but no significant association between dementia and the risk of wrist or vertebral fracture, even in patients with osteoporosis [39]. Conversely, other studies have raised the possibility of bidirectionality in the association between fractures and dementia. A study within Taiwan’s health insurance system recruited over 66,500 patients with fractures and over 133,500 control subjects without fractures, matched in terms of age, sex, and index year and then followed up for 12 years. The study demonstrated that the overall incidence rate of dementia in individuals with fractures was 41% higher than that in individuals without fractures (6.05 vs 4.30 per 1000 person-years) at an adjusted hazard ratio of 1.38 (95% confidence interval 1.32–1.45) after adjustment for age, sex, urbanization, and individual disorders or comorbidities [42].

Community-based prospective cohorts such as the Medical Research Council (MRC) National Study of Health and Development, the Framingham Study and the Study of Osteoporotic Fractures have demonstrated associations between BMD and cognitive ability or decline [46–49], brain volume [50], and incidence of AD [51]. In an early (2005) Framingham study of 987 participants with BMD measures, and a follow up time of 8.3 years, women in the lowest quartile of BMD had double the risk of AD; however, when men were considered alone, there was no significant increase in risk of AD [51]. A 2023 meta-analysis of 10 studies built upon this evidence, demonstrating that patients with cognitive impairment had an increased risk of BMD-defined osteoporosis (relative risk 1.56 any cognitive impairment, and 1.70 (95% CI 1.23–2.37) for AD), though heterogeneity between studies was identified due to problems with cognition classifications, sex differences, variation in global regions and study designs [52].

A recent well-designed meta-analysis of three studies (Framingham Heart study, Rotterdam Study and the Memory and Ageing Project, n = 4431 with 606 incident dementia diagnoses) asked the question whether baseline BMD alone, or greater BMD loss, were associated with greater risks of incident dementia or AD, important when considering each as a potential biomarker and when examining potential pathological mechanisms connecting bone loss and cognitive decline. They measured the annualised change in BMD calculated from repeated measures and found that, whilst higher BMD was protective against incident dementia (hazard ratio 0.91 per SD increase (95% CI 0.84–0.995)), prior bone loss was only significantly associated with dementia incidence in one of the three cohorts, Framingham [53].

Whilst associations between dementia and fractures may in part reflect shared risk factors, there are likely to be direct causal relationships, possibly bidirectionally [54] or mediated through other factors such as muscle loss [55, 56]. In the English Longitudinal Study on Ageing, the presence of MCI was shown to be associated with a higher incidence of sarcopenia at ten-years follow-up (OR 1.24, 95% CI 1.09–1.60 in a multivariate analysis), demonstrating a likely role of MCI as a predictor of the onset of sarcopenia in older people [57]. Osteosarcopenia, the combination of osteoporosis and sarcopenia [58], is associated with cognitive impairment, due to similar risk factors including genetics, endocrine function and mechanical factors, in addition to the suppression of nutrition and appetite (and hence lower protein intake). In a Taiwanese community care study, where DXA and comprehensive geriatric assessment were performed on 337 participants, the prevalence of cognitive impairment was found to be greatest in those with sarcopenia (40%), followed by osteosarcopenia (35%) versus those with normal BMD and muscle mass, strength and function [59].

Further reinforcing the need for fracture prevention in this population, there is evidence that mortality following a hip fracture is greater in patients with AD. In a retrospective UK study of over 18,000 participants, patients with AD had a 3.2 times increased mortality risk (HR 3.2, 95% CI 2.4–4.2) than non-AD patients following a hip fracture, this hazard was of similar magnitude to having heart failure in combination with a hip fracture [60]. In a meta-analysis of an “oldest old” population of centenarians, dementia was the most common comorbidity in those patients who died following a hip fracture (26.2%, followed by hypertension 15.6%), with a very high 1 year mortality of 53.8% (95% CI 47.2 to 60.3%) and an in-hospital mortality rate of 14.1% [61], emphasising the frailty, low resilience and low probability of post-fracture recovery in this population.

To summarise, there is a wide range of evidence to link cognitive decline, dementia, osteoporosis and fractures, but little clarity as to whether these associations are causal or represent common risk factors and/or common underlying mechanisms. Key wider considerations include falls, poor nutrition and appetite, medication use and adherence, exercise, oestrogen exposure and sarcopenia amongst other factors, and indeed how much of the risk might be independent of the existing assessment measures such as FRAX and BMD measurement. Furthermore, current epidemiological evidence is limited largely to the United States, Europe and a few Asian and South American countries, there is very limited evidence in African populations, where osteoporosis and dementia are emerging problems [31, 62].

## Mechanistic considerations

### Common and bidirectional pathways

Many common mechanisms are proposed across a wide range of ageing-related diseases [63]. In older people, dementia and osteoporosis often occur in a multimorbidity cluster with other conditions, for example sarcopenia (loss of muscle mass, strength and function with ageing). Indeed, there is increasing evidence of underlying conditions such as immunosenescence, chronic inflammation (inflamageing), oxidative stress and altered mitochondrial bioenergetics, together with factors such as hormonal changes (including decreases in oestrogen and testosterone) and altered gut microbial flora contributing to both dementia and osteoporosis risk [64–66].

**Fig. 1:**
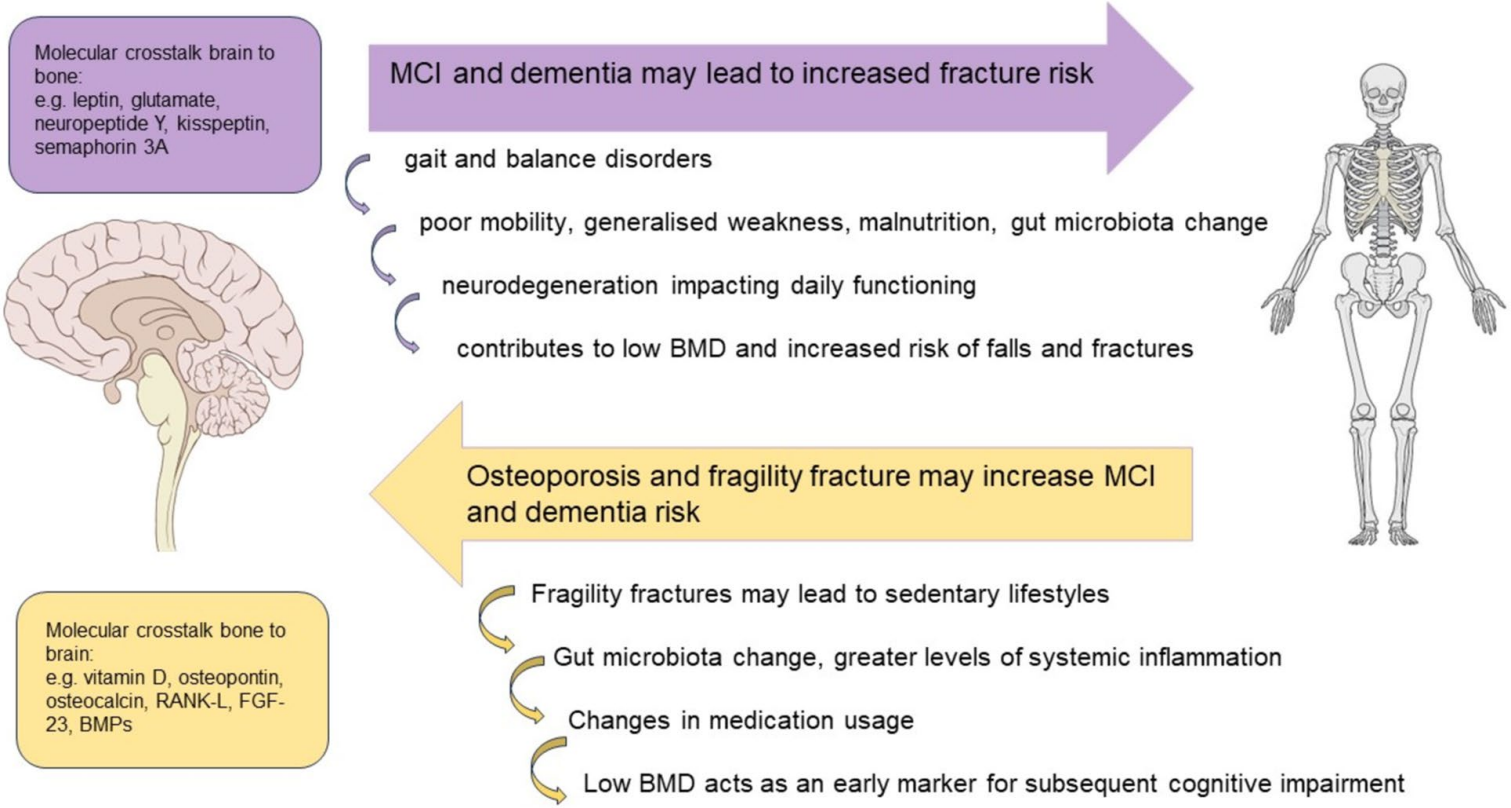
There are bidirectional relationships between cognitive impairment, dementia, fragility fractures and low BMD. Factors such as sedentary lifestyles, vitamin D deficiency and medication usage influencing both brain and bone health may contribute to the occurrence of falls, further complicating the interplay between falls, cognitive impairment and fractures. Modified from Ruggiero et al. Ageing Research Reviews 2024 [99] under the Creative Commons licence http://creativecommons.org/licenses/by/4.0/

In addition to common underlying mechanisms, related bidirectional effects have been proposed (Fig. 1). For example, whilst systemic inflammation has effects on both bone and brain, a fracture may trigger a profound inflammatory cascade, potentially contributing to neuroinflammation and the development or progression of cognitive impairment. This has been proposed in studies suggesting that there is a temporal relationship between fracture and dementia onset, with a population-based cohort study showing that fracture is an independent risk factor for dementia, and that fractures (at any site), in adults above the age of 60 years increase the risk of developing dementia within 12 years by 41%, with hip fractures conferring the greatest increase risk (60%), vertebral fractures by 47%, and thigh, leg or ankle fractures increasing the risk by 35% [42, 67]. It is possible that delirium, a sudden onset acute confusional state that occurs over a couple of hours or days, which often complicates fractures (especially of the hip), may be a contributing factor to the onset and progression to dementia [68]. Delirium is a well-recognised modifiable risk factor for dementia, which is often worse in people with poorer baseline cognitive function [69, 70].

Low BMD, without fracture, appears to be associated with cognitive impairment. In another study of 655 community dwelling older women, BMD was measured by peripheral quantitative computed tomography and cognition was assessed with the mini mental state examination (MMSE) over a 3-year period. It was found that higher cortical BMD (but not trabecular BMD) was protective against incident cognitive impairment (OR 0.93, 95% CI 0.88–0.98) and worsening cognitive performance. The authors suggested that BMD might represent an independent and early marker of subsequent cognitive impairment and that physicians should assess and monitor cognitive performance in the routine management of all the women with osteoporosis [71].

## Molecular mechanisms linking cognitive impairment and bone health

### Brain-bone signalling

The contribution of neuronal signalling to the regulation of bone remodelling and homeostasis began with a focus on leptin (an adipose tissue- derived hormone, involved in energy regulation and metabolism) [72]. Studies undertaken two decades ago in knockout mice deficient in leptin (ob/ob mice) demonstrated that these animals have high vertebral trabecular bone mass, which can be reversed by infusion of leptin into the third hypothalamic ventricle) [72–74]. Furthermore, leptin has been shown to act on human marrow stromal cells to enhance osteoblast differentiation and to inhibit adipocyte differentiation [75, 76]. It consequently became apparent that the pathways connecting bone and brain play crucial roles in both bone metabolism and energy regulation. A rapid expansion in studies working on the brain-bone interface has followed, with both the sympathetic and parasympathetic nervous systems having been shown to regulate bone through a variety of pathways. Serotonin, when produced peripherally, acts as a hormone to inhibit inflammation, but when produced in the brain, it acts as a neurotransmitter to exert a positive and dominant effect on bone mineral accrual, by enhancing bone formation and limiting bone resorption [77]. Adiponectin, parathyroid hormone, circadian genes, neuropeptide Y, muscarinic and nicotinic receptors, beta-adrenergic receptors and the innervation of bone by sensory nerves have been implicated [78–81].

### Molecular and cellular drivers

A variety of molecules and mediators have been proposed as potential molecular and cellular drivers for direct biological effects between brain and bone, as detailed in a review of the effects of a spectrum of neurological disorders on bone health (incorporating AD, Parkinson’s disease, stroke and a variety of other pathologies) [82]. For example, in AD, amyloid precursor protein (APP) and β-amyloid are increased in both neurons and osteoblasts, where they impair neuronal and osteoblast function and proliferation, and these molecules have also been shown in transgenic mice to promote the activity of osteoclasts leading to greater bone resorption [83]. Apolipoprotein E (ApoE; the ApoE4 gene is associated with atherosclerosis and Alzheimer’s disease) promotes osteogenesis and decreases osteoclastogenesis, with aged ApoE-KO mice exhibiting severe osteoporosis compared to Wild Type mice [84]. Wnt/β catenin promotes both synaptic health in the brain and osteoblast differentiation and increased bone mass, with a possible neuroprotective role of Wnt proteins in AD [85]. Triggering Receptor Expressed on Myeloid Cells-2 (TREM2), meanwhile, has been shown to protect microglia in the brain and control the rate of osteoclastogenesis [86].

A recent review of the clinical evidence suggests significant molecular crosstalk between the bone and the brain, with wide ranging bidirectional effects of the aforementioned AD pathogenic proteins, AD risk genes, neurohormones, neuropeptides, the autonomic nervous system, neurotransmitters, and brain-derived extracellular vesicles (EVs) impacting on bone cells, bone-derived proteins, bone marrow-derived cells, bone EVs, and inflammatory cascades [87]. These mechanisms give some biological plausibility to the observed epidemiological relationships.

There is thus evidence to support links between brain and bone in terms of underlying common mechanisms, but also potentially direct pathways from brain to bone. Much of this work stems from animal models and in vitro studies, with direct human relevance not well established. Further work will be needed to ascertain whether such pathways might act in human disease and thus whether these might offer the potential for therapeutic intervention.

## Gut microbiome as a link between brain and bone

### Gut microbiome: biodiversity and dysbiosis

The gut microbiome is a community of microorganisms symbiotic living with the host in the gut lumen, with advanced metagenomic studies demonstrating that between 1000–4000 different taxa can be detected in each human faecal sample. There are some bacterial taxa that are predominant (e.g. *Bacteroides, Prevotella*) and there are also a number of minor players with low representation but metabolic activity, including bacteria that are able to synthesise short chain fatty acids (e.g. *Faecalibacterium prausnitzii*). The gut microbiota exhibits considerable resilience to stressors—in young people it often returns to its previous state after antibiotic treatment—but in older people there is reduced biodiversity after a stressor such as an antibiotic course, with a higher probability of an imbalanced gut microbiome, at the cost of taxa which are important for maintaining health [88]. There is considerable inter-individual variability in the healthy gut microbiota and this variability can be explained mainly by environmental factors including diet, geographic location, age and gender, exercise and chronic drug treatments, in addition to other factors including mode of delivery, smoking, immune system function and host genetics [89]. The way we age may depend on how interactions are established with the microbial communities harboured in the gut, with frailer people shown to have reduced biodiversity.

A study in 191 older Irish subjects living in different settings (community versus nursing home dwellers) identified two completely different clusters of gut microbiota, specifically related to the place of residence, diet and physical performance, with some species over-represented in nursing home residents, and others in community dwellers. Loss of community-associated gut microbiota correlated with increased frailty [90]. Subsequent studies have shown that the way we age may at least partly depend on how the human body able to establish interactions with the microbial communities harboured in the gut. Those ageing and remaining robust may have cooperative interactions with their microbiota, whereas those developing frailty and disability are more prone to reduced biodiversity and dysbiotic microbiota, predisposing to inflammation and disease [91, 92].

The gut microbiota can modulate physiologic functions at multiple levels, particularly because it can affect inflammation, not only in the gut but also at the systemic level. It can regulate gut permeability, allowing the entry of inflammatory mediators, short chain fatty acids (SCFA) and butyrate into the systemic circulation. Through changes in immune system activation, neuro and systemic inflammation, insulin sensitivity and oxidative stress, physiologic functions can be modulated at multiple levels, not least in the brain, muscle and bone [93].

### Gut microbiome, dybiosis and dementia

There is a large body of evidence, coming mainly from mouse models of dementia, supporting the idea that there may be a gut-brain axis contributing to the pathophysiology of dementia. Greater numbers of pro-inflammatory bacterial taxa, at the expense of anti-inflammatory taxa, lead to immune system activation and local and systemic inflammation [94]. There are bacteria which promote production of bacterial amyloids, and inhibitory neurotransmitters, able to influence the risk of cognitive dysfunction. The gut microbiome also is able to influence the blood–brain barrier, and disruption of this—influenced by circulating SCFA levels—can lead to translocation of gut derived compounds into the brain contributing to the activation of microglia, neuroinflammation, amyloid and neurofibrillary tangle deposition, ultimately leading to neuronal loss [95–97].

Studies comparing gut microbiota composition between subjects with or without dementia are limited by cross-sectional design and generally small sample sizes, with very few studies from Western countries (most are from China), often not taking into account dietary factors, appetite and calorie intake which is often lower in patients with dementia. These limitations aside, such studies have shown that patients with MCI or dementia have different faecal microbiota composition than healthy controls—though there is inconsistency in the microbial biomarkers of dementia detected across the different studies [98]. A recent study of 164 subjects with preclinical AD suggests that the biomarkers of dementia in the gut microbiota change dynamically across dementia stages, from healthy, to MCI to dementia, with the authors noting different additional risk of dementia conferred by exposure to certain taxa at different times [99, 100].

### Gut microbiota and brain-bone axis

The gut microbiota may modulate bone health indirectly (modulating brain function via the gut-brain axis) or directly (whereby modulation of osteoblast and osteoclast function is mediated by microbial products) [101]. Indeed, there is evidence that a dysbiotic gut microbiota can decrease BMD via a variety of mechanisms, including the regulation of intestinal mineral absorption, oxidative stress, modulation of immune responses and anabolism [102]. Subjects who have a healthy gut microbiome may be more likely to have better bone health via multiple potential mechanisms, including the promotion of IGF-1 synthesis by the gut, the regulation of PTH anabolism by butyrate synthesis, regulation of anabolism mediated by circulating levels of SCFAs, increased bioavailability of oestrogens, regulation of CD4 cells and T-reg cells (balancing pro and anti-inflammatory cytokines), modulation of osteoclast by indole derivatives, upregulation of TLR 9, reduction of oxidative stress and upregulation of endothelial nitric oxide species [103–105].

Osteoporosis has been shown to be associated with gut microbiota alterations. A study in 2019 analysed the faecal microbiota of 181 subjects age 55 to 75 years old and identified various microbial biomarkers associated with reduced BMD [106]. Across the various studies of this nature, there is considerable inconsistency in the microbial biomarkers shown to be associated with osteopenia or osteoporosis; the largest study (n = 1776 healthy adults, 266 with femoral and 179 with lumbar spine osteoporosis) identified *Actinobacillus*, *Blautia, Oscillospira* and *Bacteroides* to be associated with poorer bone health, and identified that the microbial metabolism of tryptophan and degradation of branched chain fatty acids was associated with osteoporosis [107]. The outcome of fracture, as opposed to low BMD, was studied by one Japanese group (with a very small sample size, n = 38 postmenopausal women, 11 with a history of fragility fracture) which demonstrated that higher degrees of dysbiosis were observed in women who had fractured [108].

The most important point regarding gut dysbiosis and its regulation of bone health is the function of the bacteria, and in particular, the gut microbiota represents a fundamental regulator of tryptophan metabolism (the kynurenine pathway) and the balance between the end products, quinolinic and kynurenic acid [109]. Alterations of the kynurenine metabolic pathway at the gut microbiome level are associated with neuroinflammation and altered synaptic transmission typical of dementia, alongside altered bone homeostasis leading to osteoporosis and increased fracture risk [110]. Kynurenines have been proposed as a biomarker for osteoporosis and sarcopenia, in addition to being potential new pharmacological treatment targets [111]. These functional aspects may be the common driver linking the gut microbiota with cognitive dysfunction and poor bone health. The gut microbiota also regulates the synthesis of serotonin from tryptophan at the gut level. Gut dysbiosis promotes serotonin (5-HT) synthesis in the gut, leading to inhibition of bone formation and consequent decreases in BMD, whilst eubiosis promotes vagal nerve signalling to the brain, increasing local 5-HT synthesis, activating parasympathetic and glutamate signalling, inhibiting bone resorption and stimulating bone formation [77, 112].

This growing evidence base leads to the question whether administration of probiotics (Lactobacillus, Bacillus, Bifidobacterium) and prebiotics (inulin, galacto-oligosaccharides (GOS)) can modify gut microbiota, thereby potentially influencing cognitive and musculoskeletal health [113]. There is some evidence that these may modulate mineral absorption, tryptophan metabolism and serotonin synthesis in the gut and brain, thereby reducing inflammation and promoting skeletal anabolic functions. In clinical terms, various studies in older people with and without osteopenia and osteoporosis have suggested that pre and probiotics may reduce the pace of BMD decline and ameliorate the markers of bone resorption, and there is evidence in mice that they increase BMD and reduce fracture risk [112]. However, it should be acknowledged that the available evidence is limited to a few studies with small sample sizes; there is certainly further work to be done.

To summarise, there are common mechanisms shared by dementia and osteoporosis mediated by the age-related changes in the gut microbiome, and the gut microbiome probably influences brain functions in different ways according to different phases of dementia development. The gut microbiome can also influence bone deposition and resolution both directly and indirectly, through the mediation of brain function [serotonin and autonomic nerve signalling]. The clinical implications of these mechanisms are still unclear, particularly considering that the available therapeutic arsenal is very limited. There is a need for further large-scale intervention studies with prebiotics and probiotics, taking into account cognitive function and bone parameters as clinical endpoints in older individuals. Whilst the Mediterranean diet is shown to induce favourable changes to gut microbiome diversity [93] there is little evidence to support the use of nutritional supplements at this time. Given that the majority of studies looking at the gut microbiome, dementia and osteoporosis are cross-sectional, it is difficult to attribute causality, particularly as dementia in itself leads to anorexia and changes in dietary patterns—including reduced dietary diversity—which may have downstream effects on gut dysbiosis [114]. It is also important to consider the longitudinal effects of dietary changes in the microbiota and whether or how this can impact upon cognitive impairment, and the direction of causality implied.

## Nutritional changes, cognitive impairment and bone health

Dietary strategies in the early stages of preclinical cognitive decline or mild cognitive impairment aim to prevent or slow the decline into MCI and dementia. In this regard, maintenance of a healthy microbiota and control of cardiovascular risk factors are promoted using a nutrient dense dietary pattern such as the Mediterranean diet, are in line with WHO guidance for risk reduction of cognitive decline and dementia [115]. As dementia progresses, more targeted nutritional strategies may be required to correct nutritional deficiencies and treat protein energy malnutrition and weight loss. Undernutrition and weight loss are very common in people with cognitive impairment [116–118]; a recent meta-analysis showed that the pooled prevalence of malnutrition risk in those with dementia (with studies mainly conducted across European and South Asian populations) was 57.4% (95% CI 49.4–65.3, *p* < 0.0001, I^2^ = 97.4%) [119]. Lower nutrient levels are found in the blood of patients with dementia, with suboptimal status of B vitamins, vitamin D, E, C, and the carotenoids. Studies have suggested that up to 94% of care home residents are deficient in vitamin D, and may have reduced intake of other key nutrients including omega 3 fatty acids, calcium, selenium and vitamin K, all of which are relevant for bone and muscle health [120]. Untreated malnutrition in dementia and MCI can lead to faster functional and cognitive decline, leading to poorer mobility and loss of independence, which of course is relevant for muscle and bone health [121].

**Fig. 2:**
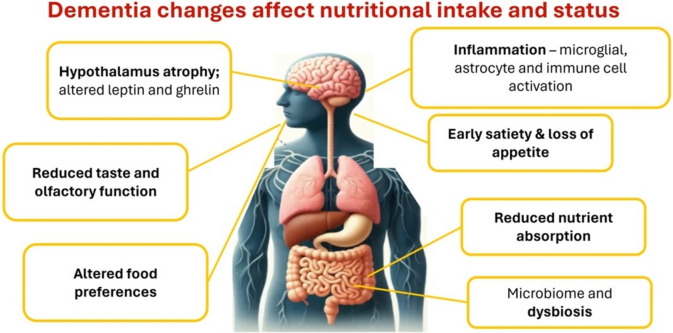
Dementia affects appetite regulation, nutritional intake and absorption. Created with Biorender

The mechanisms underlying weight loss in cognitive impairment are multifactorial. Preclinical dementia changes cause neuroinflammation and hypothalamus atrophy (which can alter hypothalamic hormones ghrelin and leptin) leading to early satiety and loss of appetite. Sensory function may also be affected, leading to reduced taste and smell. These non-cognitive changes are observed prior to the onset of cognitive impairment and may lead to altered food preferences, reduced food intake and undernutrition. Nutritional status can also be compromised by changes in the gut microbiome as previously discussed, reducing the bioavailability of nutrients (Fig. 2). In people with cognitive decline there may be deterioration in the ability to shop, cook, prepare and eat meals. People living with advanced dementia can forget to eat and drink, while the loss of motor function can lead to dysphagia, often necessitating a texture modified diet, making it difficult to meet nutritional requirements. Psychological symptoms of apathy, sadness and depression can lead to loss of interest in food—and some psychotropic drugs can reduce appetite. Additionally, behavioural symptoms such as sleep disturbance, wandering, restlessness, overactivity can lead to increased energy expenditure, so it can be difficult to meet energy demands to maintain body weight.

It is not clear whether weight loss is part of cognitive decline or a prodrome. In a meta-analysis using prospective data from 2.8 million adults (57,000 with dementia), having a low BMI (under 18.5 kg/m^2^) in earlier adult life was associated with a 26% elevated risk of all-cause dementia (HR 1.26, 95% CI 1.20, 1.31). Weight loss was associated with around a 30% increased dementia risk and has been observed 1–2 decades prior to the onset of cognitive impairment [122]. Of course, these observations are likely due to reverse causality, nevertheless it remains unknown whether addressing undernutrition early in the disease course can delay cognitive impairment and dementia [122]. Given that low BMI in older age and midlife obesity appear to increase dementia risk an important public health message is to maintain a healthy body weight over the lifecourse [123]. The European PROMED-COG consortium are working to increase knowledge on the balance between the benefits of a protein- enriched Mediterranean diet and/or physical activity for prevention of undernutrition, and promotion of healthy cognitive ageing, with a current clinical trial in older adults with subjective memory decline ongoing [124, 125].

While there are few data on diet intervention for promoting bone health in dementia, increasing dairy foods may be important. A cluster randomised controlled trial of Australian care homes involving over 700 vitamin D-sufficient adults in their 80 s—over half of whom were cognitively impaired—showed that increasing dairy food reduced the risk of falls and fractures. The 2-year study demonstrated that increasing dairy food (milk, yoghurt and cheese) from 2 to 3.5 servings per day (mean 1142 mg/day calcium, 69 g protein), versus fewer than 2 in the controls (mean 700 mg/day calcium, 58 g protein), reduced risk of all fractures by 33% (HR 0.67 (95% CI 0.48–0.93), with an impressive 46% reduction in hip fractures (HR 0.54 (95% CI 0.35–0.3) and an 11% reduction in falls (HR 0.89 (95% CI 0.78–0.98), with a significant decrease in hip fractures and falls at 3 months and 5 months respectively. Therefore, a simple, readily accessible intervention in a care home setting in people with cognitive impairment might have important benefits on musculoskeletal health [126].

To conclude, epidemiological data indicate that cognitive impairment is associated with suboptimal nutrient intakes and that correcting nutrient deficiencies is important for bone health. A nutrient dense diet, without supplementation, can provide the necessary nutrients, and the Mediterranean diet has been shown also to have favourable effects on osteoporosis risk. Nutrient deficiencies should be treated in those who are deficient, but replacement of single nutrients has not been shown to improve cognition or prevent dementia progression. Routine supplementation of vitamin D in daily low dose regimens (as opposed to high dose boluses) to maintain sufficiency (serum 25(OH)D > 50 nmol/l) is important, combined with calcium where needed, to reduce fractures and possibly to reduce AD risk in older populations [127–129]. Despite these findings from observational studies, a recent two-sample Mendelian Randomisation study found no clear evidence to support a protective role of increased vitamin D concentrations on cognitive performance in individuals of European ancestry [130].

In terms of research gaps, whilst it is evident that undernutrition and weight loss can manifest early in preclinical dementia—and can accelerate functional and cognitive declines—the impact on bone health is not known. Little is known about optimal diet and nutritional strategies aimed at maintaining or improving brain and bone health at different stages of cognitive impairment, and it is likely that the timing of interventions is particularly important. However, these are long term strategies and the ability to support sustained behaviour changes in patients and their care givers is needed, taking into account the setting—care home versus free living populations. Whilst there is a gradual accumulation of brain histomorphometric changes of AD with age, then it is important to consider which targeted, well-timed interventions should be recommended to maximise benefit and minimise cost.

## Medications for dementia and their impact on bone health

There is evidence that medications used in dementia may contribute to the occurrence of falls, potentially resulting in fractures. In preclinical trials, the administration of donepezil, a commonly used dementia drug (a reversible inhibitor of anticholinesterase (AChEI) indicated for mild to moderate dementia in AD), affects energy metabolism and favours bone mass accrual in healthy young wild type mice [131]. There is also evidence that donepezil improves bone quality by reducing the number of bone resorbing osteoclasts [131].

On the other hand, memantine (a glutamate receptor antagonist, licenced for moderate to severe dementia in AD) had mildly unfavourable effects on the skeletal system of female rats with normal oestrogen levels [132]. However, the skeletal effects were oestrogen dependent: there was no effect of memantine in oestrogen deficient rats and the potential unfavourable skeletal effects of memantine may be less pronounced in oestrogen deficient post-menopausal women than in premenopausal women; these unfavourable effects have not been reported in humans [132].

There may be an important interplay between these drugs and fracture risk through impacts on falls and syncope in patients with cognitive impairment. A recent systematic review of all randomised controlled trials of cholinesterase inhibitors (donepezil, galantamine and rivastigmine) covering 53 studies and over 25,000 patients showed that cholinesterase inhibitors compared to placebo were associated with a reduced risk of falls (risk ratio [RR] 0.84; 95% CI 0.73–0.96, p = 0.009), but an increased risk of syncope (RR 1.50; 1.02–2.21, p = 0.04) and were not associated with an increased risk of fractures [133]. Syncope arises as a rare side effect of these drugs and represents the mechanism underlying just a small proportion of falls. In fact, a Cochrane systematic review on memantine found no difference in falls between memantine and placebo groups [134].

There are some observational studies in this area, including a nested case–control study using the UK CPRD which suggests that any past use of AChEIs—at least two prescriptions—is associated with a reduction in the fracture risk (adjusted OR 0.80; 95% CI 0.71–0.91), with better adherence strengthening the association [135]. Of course, there are confounding factors which cannot be accounted for when comparing users of a medication with non-users, as drug prescription is not a random behaviour and the populations are by definition different (confounding by indication).

In another cohort of male US veterans with dementia without a prior history of fractures (n = 360,000), the hazard of any fracture among AChEI users was compared with those on other or no dementia medications. The fracture hazard was significantly lower in the fully adjusted Cox proportional hazards models (HR = 0.81; 95% CI 0.75–0.88) [136]. The evidence, however, is not wholly reassuring regarding the link between ACHEI use and fracture; a Korean insurance database study suggested the opposite, that the use of AChEIs in patients with AD was associated with an increased risk of osteoporotic fractures (adjusted OR 1.18; 95% confidence interval 1.07–1.31). The authors propose some explanations for this discrepancy, perhaps there may be population based differences [137]. Interestingly, a Japanese retrospective study of over 300,000 patients with AD, using propensity score matching, showed that in patients with AD with dementia medication use (AChEIs and memantine) compared with AD patients without dementia medication use, the incidence of hip fractures (1.9% vs. 4.0%, p < 0.001) and all clinical fractures (9.0% vs. 10.5%, p < 0.001) significantly decreased, but that of radial fractures increased (1.0% vs. 0.6%, p < 0.001). Memantine, specifically was associated with a decreased rate of vertebral fractures indicating a difference from the AChEIs in the mechanism of action potentially underlying these associations [138]. A meta-analysis published in 2024 using data from seven observational studies showed that the risk of fracture was not statistically different between dementia patients who received acetylcholinesterase inhibitors and those who did not receive them (OR 1.44; CI 0.95–2.19, *P* = 0.09) but that when used for periods of more than two years they may have a protective role in reducing fracture risk [139].

To conclude, most studies suggest that dementia drugs, particularly AChEIs, have non-harmful and possibly a potential beneficial effect in reducing the risk of falls (seen in both RCTs and observational studies) and fractures (in observational studies) in older adults with dementia. Controlling for confounders—particularly confounding by indication, as many of these drugs could be prescribed at an earlier stage of dementia, and possibly in individuals with better mobility—is a problem in many of the aforementioned studies. Proper benefit-risk balance assessment of each treatment is important, as is avoiding drug-drug interactions in older adults with dementia. Recently, the role of menopausal hormonal therapy (MHT) in the onset and progression of dementia has become a topic of relevance due to different studies showing beneficial, neutral, or harmful effects [140]. The beneficial effects of oestrogens on bone are widely known, current literature suggests robust anti-fracture efficacy of MHT in patients unselected for low BMD, but a careful benefit-risk balance needs to be struck regarding the timing and duration of use [141]. There is an evident need for more research and guidelines on pharmaceutical choices in patients with dementia and cognitive impairment.

## Cognitive impairment and reduced adherence to osteoporosis medications

Non-adherence to oral treatments prescribed for osteoporosis is a widespread problem and is associated with an increased risk of fractures. Few interventions have been shown to improve adherence or persistence, and recommendations for clinicians to help improve adherence in their patients have been proposed by a previous ESCEO working group (2019) [142]. Multiple factors determine a patient’s likely adherence—these include patient related factors such as age, sex, educational level, living alone, and cognitive function, treatment related factors such as polypharmacy and adverse reactions, health system related factors such as lack of counselling on the medications, numbers of practitioners and caregivers, and socioeconomic factors such as drug costs and insurance coverage [142].

Cognitive impairment has been identified as a risk factor for medication nonadherence in various countries, including a cross sectional study of patients over the age of 65 in 16 Chinese hospitals (n = 773 participants), which showed that overall almost 32% of patients studied were not adhering to their medication [143]. A Shanghai primary care study (n = 436 patients over 60 years with chronic diseases) showed, again, that medication adherence was poor in almost half of patients, and that MCI was associated with poorer medication adherence (OR 2.64, 95% CI 1.64–4.24 for nonadherence in MCI patients versus no MCI) [144]. In Oregon, USA, a study in 38 “healthy” subjects over the age of 65 was undertaken (without a diagnosis of MCI or dementia), medication adherence was tracked with automated pillboxes, and the included subjects underwent cognitive testing. The study showed that even participants with very minimal cognitive impairment had lower adherence, with 72% of subjects with lower cognitive function having poor adherence, versus 25% in the normal cognitive function group, particularly in medications which were prescribed to be taken in the evening [145].

In studies focusing on osteoporosis medication, in general, cognitive impairment has been shown to reduce adherence and persistence. In a Spanish study of 4856 patients using dispensing data, non-persistence (discontinuation for more than 90 days) was seen more commonly in patients with dementia (HR for non-persistence 1.18 (95% CI 1.02–1.38) in treatment naïve, and 1.31 (95% CI 1.13–1.53) in experienced users) [146]. A Canadian study using dispensing data showed a similar picture; in over 39,000 patients with osteoporosis, a third of whom had a diagnosis of dementia, osteoporosis drug dispensation occurred almost half as often in patients with dementia, compared to patients without (adjusted OR 0.55, 95% CI 0.44–0.69) [147]. A US Medicare study (n > 41,000) showed a similar pattern in prescription rates, with patients with AD receiving osteoporosis drugs at a lower rate than their non-AD counterparts, a difference which was even more pronounced in nursing home residents and subjects over the age of 85 [148]. In the oldest-old (patients over the age of 90 years) in Singapore, cognitive impairment was also associated with a lower odds of starting an osteoporosis treatment (OR 0.25, 95% CI 0.07–0.83) [149].

In summary, non-adherence to oral treatments is prevalent in older patients in general and is multifactorial in origin—as is underdiagnosis and undertreatment—and is even more prevalent in subjects with MCI and dementia. Tailored interventions in this population aimed at improving adherence may include: medication reviews, simplifying drug regimens, improved patient education, encouraging single point of care to reduce the number of prescribing physicians and the frequency of regimen changes, taking into account patient preferences of drug formulations, and automated medication reminders. Prescriber-level interventions are also vital and would aim to enhance treatment initiation in this largely older population, and to improve recognition of MCI and dementia as risk factors for fractures. Consideration should be given to choosing parenteral treatment when indicated in those who are unable to adhere to an oral osteoporosis treatment—though calcium and vitamin D is usually given alongside intravenous and subcutaneous treatments. Caregiver involvement in medication administration and adherence is key, especially for cognitively impaired and dependent patients.

## Cognitive impairment and its association with falls

Falls are a geriatric syndrome, with a variety of intrinsic and extrinsic factors increasing the risk of falls, including dementia, ageing, impaired vision and hearing, polypharmacy, gait impairment and orthostatic hypotension. Older adults with dementia fall two to three times more frequently than older adults without dementia. A recent systematic review suggested that the prevalence of falling was 43% for patients with MCI (over the age of 50 years) and that risk factors for falls included slow gait, dual-tasking, poor postural control and the non-amnesic type of MCI [150]. There is a particular pre-dementia condition, the motoric cognitive risk syndrome, which is associated with the simultaneous reduction of memory performance and motor activity, and an increased risk of vascular dysfunction (cardiovascular disease, hypertension, diffuse vascular lesions) which can progress to vascular dementia and is particularly linked with an increased risk of falls [151]. Different conditions underlying cognitive impairment (e.g. AD, dementia with Lewy Bodies, Parkinson’s disease, vascular dementia) can all impact upon falls risk via different mechanisms (e.g. orthostatic hypotension, visual hallucinations, dyskinesia and other sensory impairments) [152–155].

There appears to be a significant difference in the risks of falls between institution-dwelling older adults with dementia and community dwellers, with particular differences in psychosocial risk factors between the groups (e.g. verbally disruptive or attention seeking behaviour, depression, distress in caregivers and the severity of dementia) [156]. Unsurprisingly, those who fall have poorer mobility and slower gait speed, but interestingly, in a meta-analysis, reducing global cognition was not associated with falls, suggesting that, in terms of falls reduction strategies, interventions targeting balance impairment rather than cognition might be more fruitful, which is in line with current clinical recommendations and practice [157].

It is important to remember that in general, polypharmacy increases risk for falls and fractures and that drug-drug interactions can increase the complexity of patient assessment. A European expert group recently convened to support clinicians in the deprescribing of fall-risk inducing drugs (FRIDs) by designing a screening tool “STOPPFall (Screening Tool of Older Persons Prescriptions in older adults with high fall risk)”, based on evidence from meta-analyses and national fall prevention guidelines in Europe. Fourteen medication classes (mostly psychotropic medications, but also other drugs like medications for pain, hypertension and overactive bladder) were included in the list of FRIDs and the effectiveness of such an intervention will be evaluated in future studies. No osteoporosis drugs were listed as FRIDs [158]. Psychotropic pharmacotherapy (e.g. antipsychotics, benzodiazepines, SSRIs), opioids and drugs with anticholinergic burden should particularly be avoided in patients at high falls risk [159–161]. Anticholinergic drugs are numerous (and vary in potency) and are often prescribed in combination, with falls risk greatly elevated when even moderate potency anticholinergic drugs are combined. Prescribers should consider, when prescribing for older adults with MCI or dementia, whether each additional medication is worth the increased risk of falls [161].

Caregivers can also play a role in the mitigation of falls in this population, particularly as an important risk factor for falls is the patient’ home environment (e.g. lighting, stairs, trip hazards), footwear and assistive devices [162]. Caregivers can be responsible for seeking and coordinating care (e.g. facilitating participation of older adults in community exercise programmes, dietary education and supplementation), and can help to engage in medication management and other activities (e.g. time outdoors, music, recreation) which can help to improve wellbeing [163].

To conclude, MCI and dementia are related to an increased risk of falls through various mechanisms. Direct factors (cognitive decline, balance deficits, poor mobility, slow gait speed, visuospatial deficits) and drugs (particularly antipsychotics, antidepressants, benzodiazepines, opioids, anticholinergic drugs) may impact upon falls risk. It is a research challenge to disentangle the relative contribution of each factor to the fall risk, and when applying this knowledge, a whole person approach is needed. An example of this may be that by counteracting depression, the risk of falls may be increased as the patient becomes more mobile, but their increased mobility may bring them a better quality of life and help to maintain muscle strength. Similarly, certain anti-depressants may stimulate appetite in older people with cognitive impairment, helping to counteract weight loss and nutritional deficiencies, but these drugs may have an associated falls risk [164]. Many associations are observed between different medications and drugs, without direct evidence of causality or ability to compare the magnitude of effects, which makes issuing practical guidance a challenge.

Proper benefit-risk balance assessment of each drug treatment is important, as is avoiding drug-drug interactions in older adults with dementia. There is an evident need for more research and guidelines on pharmaceutical choices in patients with dementia or cognitive impairment. It is important to ensure that when de-prescribing in older people at high risk of falls, antiresorptive drugs such as bisphosphonates are not removed from a patient’s prescription with the same stroke of the pen as antidepressants or anticholinergics.

Questionnaires and protocols for assessing and tackling falls risk are available (e.g. the Centers for Disease Control and Prevention's Stopping Elderly Accidents, Deaths, and Injuries (STEADI) initiative to help primary care providers (PCPs) and caregivers to identify and manage fall risk), but are not widely used and could simplify the approach to modifiable risks [165]. A wide range of barriers must be overcome for a patient to adopt fall prevention behaviours (e.g. denial of falls risk, self-blame, fear of falling, poor health, sedentary habits, accessibility or transport issues and lack of support or interest by health professionals). The psychological impact of caring for an older person who falls are not to be underestimated (e.g. higher levels of depression and anxiety and self-blame for not monitoring their care recipient closely enough) and support for caregivers is vital.

## The impact of physical activity and exercise on cognitive impairment and bone health

It is important, first, to consider the differences in the definitions of physical activity (PA) and exercise: PA has been defined as any body movement produced by skeletal muscle that involves energy expenditure, whilst exercise describes a subset of planned, structured and repetitive physical activity, and has, as its ultimate goal, the improvement or maintenance of physical fitness. The impact of PA on various aspects of health has been reported in observational studies, whilst the impact of exercise is generally evaluated via randomised controlled trials. A few years ago, the WHO and the International Conference on Frailty and Sarcopenia Research issued guidelines on PA and sedentary behaviour, with specific recommendations for older adults and those with chronic health conditions [166, 167].

It is widely known that being physically active is a key factor in maintaining health and the normal functioning of physiological systems across the lifecourse. The benefits of PA on bone health (both directly, and indirectly through reducing the risk of falls) have been extensively researched in both observational studies and RCTs, with varied outcomes: higher levels of PA (primarily leisure time activity or moderate or vigorous PA), particularly if undertaken regularly, are associated with up to 40% lower risk of hip and all fractures [168–173]. In experimental exercise trials, conducted in the context of additive benefits above pharmacotherapy, it is not clear whether exercise has an additional benefit (on BMD and bone turnover markers) above osteoporosis drug treatments, and no data are available on fracture outcomes, or fracture healing [174]. The link between PA and muscle health is a topic which has been reviewed by previous ESCEO working groups [175].

Physical inactivity is associated with an increased risk of premature all-cause mortality. Physically active older adults have improved functional capacity: physical and cognitive function, mobility and quality of life, and reduced musculoskeletal pain, lower risks of falls and fractures and depression. PA has important benefits on brain structure and function, and cognitive, perceptual and motor skills [167, 176, 177]. The protective role of PA against AD has been widely demonstrated, as summarized in a 2023 umbrella review [178]. The evidence demonstrating the positive effects of PA on AD risk were strongest (compared to other types of dementia), but there were also meta-analyses revealing the positive effects of exercise on cognitive function, physical performance and functional independence [178]. Whilst the evidence for such benefits is reasonably strong, due to multiple different trial methodologies there has been little consensus on the precise recommendations or guidance on PA in older people, specifically those with, or at high risk of, cognitive disorders.

**Fig. 3:**
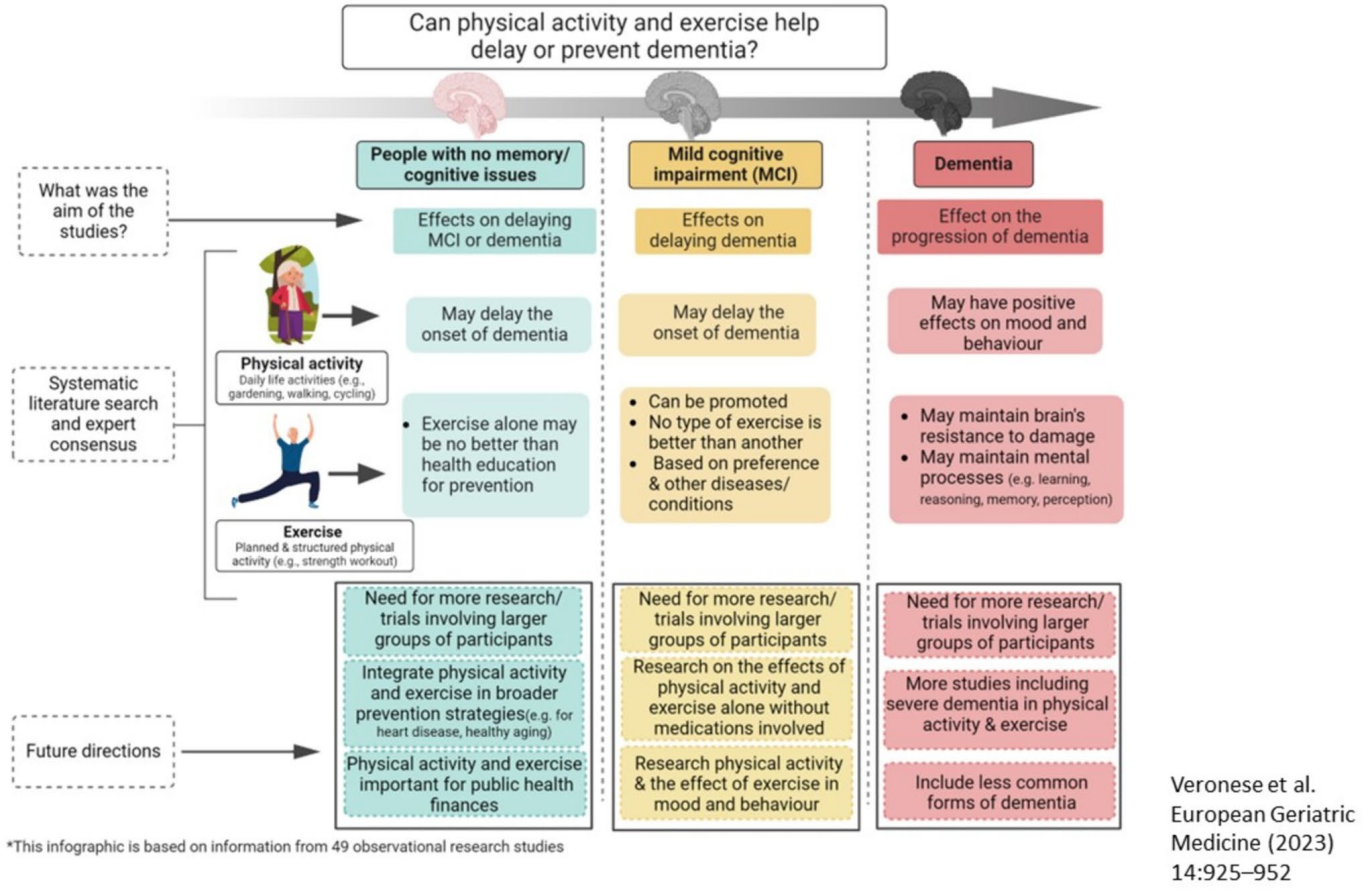
Infographic regarding the effect of physical activity and exercise in people without cognitive impairment, in mild cognitive impairment and in dementia. Created with Biorender. This figure was reproduced from Veronese et al. European Geriatric Medicine (2023) [177] under the Creative Commons licence http://creativecommons.org/licenses/by/4.0/

In terms of dementia prevention, a research question is whether, in people without dementia or MCI, physical activity and or exercise can delay the onset of these conditions. A collaborative international guideline (2023), bringing together many societies, provided a set of evidence- and expert consensus-based prevention and management recommendations (using GRADE methodology) regarding PA and exercise, applicable to older adults. The group concluded that PA may be considered for the primary prevention of dementia, reducing the risk by around 20% (any dementia, AD and vascular dementia), but in people with MCI there is continued uncertainty about the role of PA in slowing the conversion to dementia, with mind–body interventions having the greatest supporting evidence. They found that in people with moderate dementia (but not normal cognition or MCI), physical exercise may be used for maintaining disability and cognition, but all recommendations (concerning both PA and exercise) were based on a very low/low certainty of evidence. An infographic demonstrating the effect of PA and exercise in people without cognitive impairment, with MCI and with dementia is shown in Fig. 3 [179].

Since the publication of the 2023 guideline, a recent meta-analysis of 104 studies with over 340,000 participants, also showed that PA was associated with a small decreased incidence of cognitive impairment or decline (pooled RR, 0.97, 95% CI 0.97–0.99), but there was no significant assoiciation between PA and cognitive impairment in follow-ups longer than 10 years and there was no evident dose–response relationship. The specific cognitive domains associated with reduced physical activity were episodic memory (standardized regression coefficient, 0.03; 95% CI 0.02–0.04) and verbal fluency (standardized regression coefficient, 0.05; 95% CI 0.03–0.08). The associations observed were very weak and high quality studies of physical activity and cognition in midlife were scarce—most looked in older age groups and many did not have baseline measures of cognition available, and study-level measures of physical activity were imprecise. However, even weak associations may have clinically significant impacts from a population health perspective, but there are few high quality studies with follow-up beyond 10 years [180].

It is reasonable to conclude that increasing physical activity and exercise is likely to be of benefit in terms of preventing MCI and dementia and slowing physical and cognitive declines and is also likely to benefit skeletal health. PA can also improve balance and thus reducing falls and secondary fractures. There is a need for adequately powered randomised controlled trials evaluating the effect of PA and exercise for the primary prevention of MCI and dementia, and for the prevention of fracture, particularly using multicomponent (e.g. PA combined with nutritional) interventions [125, 179].

## Conclusions

There is a wide range of evidence underpinning associations between MCI and dementia on the one hand, and osteoporosis and fractures on the other, with a multitude of different mechanisms and factors at play. Bidirectional associations are present, and causal mechanisms are challenging to define due to a variety of shared risk factors. It is important to differentiate associations between MCI or dementia and BMD, as opposed to association with fracture, and other common factors such as falls risk need to be taken into account.

In this working group report a wide range of areas of scientific interest have been discussed—the epidemiology of the links between MCI and dementia and fractures and low BMD, molecular mechanisms common to both brain and bone (signalling and molecular and cellular drivers), the influence of the gut microbiome, medications (including effects of dementia drugs on bone, and the difficulties of adherence in patients with dementia and MCI), diet, nutritional status, physical activity and exercise (also linked to falls and BMD and fractures).

It is apparent that fracture risk assessment approaches used currently, such as FRAX, may not capture the additional risk of fracture associated with MCI and dementia, and further work to establish the additional risk, independent of other risk factors, is needed. Further work is also needed in the medical community to elevate our recognition of the additional fracture risk experienced by patients with MCI or dementia, and to encourage osteoporosis drug prescribing, medication adherence, nutrition and exercise guidance and support for carers, all of which should be done in the context of the MDT. Cross speciality working (e.g. primary care physicians, geriatricians, rheumatologists, neurologists, psychiatrists, endocrinologists, dieticians) working collaboratively, rather than in silos, will help to improve care of these patients—for example when prescribing medications which may be detrimental to bone health or may increase falls risk. Patients should be offered the same access to fracture risk assessment and treatment regardless of age or cognitive problems. In the context of comprehensive geriatric assessments, fracture risk assessment should be undertaken in parallel with cognitive testing- it is in patients with MCI or dementia that fractures can be most devastating in terms of morbidity and mortality.

## Research gaps

This document delineates several research gaps that warrant further investigation. First, there is a need for a global understanding of the epidemiological associations between MCI or dementia and bone mineral density (BMD) and fractures, particularly in lower-middle-income countries. There is a call for more refined risk assessment approaches in populations with MCI and dementia, particularly regarding whether adjustments to FRAX^®^ probability should be made based on the degree of cognitive impairment or its rate of progression.

Moreover, there is a recommendation to consider BMD and fractures as secondary endpoints in trials for novel dementia therapeutics, including those targeting β amyloid. Further research is necessary to explore interventions aimed at improving the gut microbiota and understanding its impact on cognition, ensuring a comprehensive characterization of participants—including factors like diet, falls risk and physical activity—to reduce potential for potential reverse causation. Underlying this, it is vital to understand how MCI and dementia influence dietary preferences and the impact of this upon the timing of maximal bone loss throughout the disease trajectory, in order to develop targeted interventions.

Investigation of practical methods to enhance adherence to osteoporosis medications in individuals with MCI or dementia is needed, including studies that assess the advantages of injectable or intravenous medications over oral agents. Lastly, a better understanding of how deprescribing policies affect the prescription of osteoporosis drugs is crucial, along with clear communication to the medical community that these medications should generally be continued in patients with MCI or dementia (unless at the very end of life), as they are at a heightened risk of fractures.

## Data Availability

No datasets were generated or analysed during the current study.
